# Synergistic Effect of Two Nanotechnologies Enhances the Protective Capacity of the *Theileria parva* Sporozoite p67C Antigen in Cattle

**DOI:** 10.4049/jimmunol.2000442

**Published:** 2021-01-08

**Authors:** Anna Lacasta, Karishma T. Mody, Ine De Goeyse, Chengzhong Yu, Jun Zhang, James Nyagwange, Stephen Mwalimu, Elias Awino, Rosemary Saya, Thomas Njoroge, Robert Muriuki, Nicholas Ndiwa, Elisabeth Jane Poole, Bing Zhang, Antonino Cavallaro, Timothy J. Mahony, Lucilla Steinaa, Neena Mitter, Vishvanath Nene

**Affiliations:** * Animal and Human Health Program, International Livestock Research Institute, Nairobi 00100, Kenya;; † Queensland Alliance for Agriculture and Food Innovation, The University of Queensland, Brisbane, Queensland 4072, Australia;; ‡ Enzootic, Vector-borne and Bee Diseases, Sciensano, 1180 Brussels, Belgium;; § Department of Biomedical Sciences, Institute of Tropical Medicine, 2000 Antwerp, Belgium;; ¶ Australian Institute for Bioengineering and Nanotechnology, The University of Queensland, Brisbane, Queensland 4072, Australia;; ‖ Research Methods Group, International Livestock Research Institute, Nairobi 00100, Kenya; and; # Department of Agriculture and Fisheries, Brisbane, Queensland 4102, Australia

## Abstract

East Coast fever (ECF), caused by *Theileria parva*, is the most important tick-borne disease of cattle in sub-Saharan Africa. Practical disadvantages associated with the currently used live-parasite vaccine could be overcome by subunit vaccines. An 80-aa polypeptide derived from the C-terminal portion of p67, a sporozoite surface Ag and target of neutralizing Abs, was the focus of the efforts on subunit vaccines against ECF and subjected to several vaccine trials with very promising results. However, the vaccination regimen was far from optimized, involving three inoculations of 450 μg of soluble p67C (s-p67C) Ag formulated in the Seppic adjuvant Montanide ISA 206 VG. Hence, an improved formulation of this polypeptide Ag is needed. In this study, we report on two nanotechnologies that enhance the bovine immune responses to p67C. Individually, HBcAg-p67C (chimeric hepatitis B core Ag virus-like particles displaying p67C) and silica vesicle (SV)–p67C (s-p67C adsorbed to SV-140-C_18_, octadecyl-modified SVs) adjuvanted with ISA 206 VG primed strong Ab and T cell responses to p67C in cattle, respectively. Coimmunization of cattle (*Bos taurus*) with HBcAg-p67C and SV-p67C resulted in stimulation of both high Ab titers and CD4 T cell response to p67C, leading to the highest subunit vaccine efficacy we have achieved to date with the p67C immunogen. These results offer the much-needed research depth on the innovative platforms for developing effective novel protein-based bovine vaccines to further the advancement.

## Key Points

- Multimerization of p67C Ag as nanoparticle increases its immunogenicity.
- Vaccine efficacy of p67C Ag increases delivered as nanoparticles.

## Visual Abstract

**Figure:**
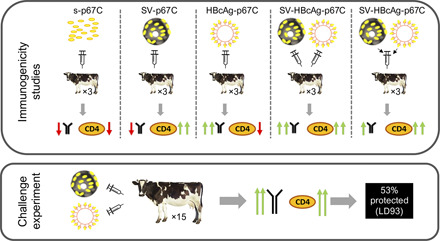

## Introduction

East Coast fever (ECF) is a lethal disease of cattle affecting 12 countries in sub-Saharan Africa. The disease, caused by a tick-transmitted apicomplexan organism called *Theileria parva*, represents a significant constraint to increasing livestock productivity in smallholder and pastoral farming systems (1). A virulent, live sporozoite vaccine based on an “infection and treatment method,” referred to as “ITM,” is commercially available but it is difficult to manufacture, it requires oversight and expertise for its use, and it is expensive (2). Hence, several laboratories are working toward development of ECF subunit vaccines. Although a classical CTL response to schizont-infected cells plays a major role in mediating immunity to ECF (3–5), we and others have previously demonstrated that immune responses to p67, the major surface stage–specific Ag of sporozoites, can contribute to protective immunity (6, 7).

Full-length recombinant p67 protein and fragments of it have been the subject of several experimental vaccine trials (reviewed in Ref. 8). Current efforts are focused on an 80-aa polypeptide sequence from the C-terminal end of p67, referred to as p67C. It contains two nonoverlapping epitopes recognized by murine sporozoite-neutralizing mAbs (9). Remarkably, an immunization regimen involving three inoculations of 450 μg of soluble p67C (s-p67C) Ag formulated in the Seppic adjuvant Montanide ISA 206 VG primes immunity to ECF in ∼50% of vaccinated cattle against an LD_70_ sporozoite challenge (7), an efficacy level that is equivalent to that primed by p67 (7, 10). However, reducing the number of inoculations of p67C to two resulted in immunity to ECF in only ∼25% of vaccinated cattle (11). Hence, an improved formulation of this polypeptide Ag is needed. With this objective in mind, we have explored improving the immune responses to p67C by changing its presentation to the bovine immune system from a monomeric soluble Ag to a particulate multimeric one by using two nanoparticle platform technologies.

The first platform exploits the ability of the core Ag of hepatitis B virus (HBcAg) to fold into chimeric icosahedral-shaped virus-like particles (VLPs) following insertion of heterologous Ag sequences into the domain between aa 76 and 81 of HBcAg. This results in the display of foreign Ags at the top of a spike structure called the major immunodominant region at the surface of the VLP in a regular array that can translate into superior stimulation of Ag-specific Ab responses (12). Exploitation of this system as a generic Ag delivery system has, however, been hampered by finding that HBcAg generally permits insertion of short polypeptide sequences (≤100 residues) as longer sequences often disrupt dimerization of HBcAg monomers and subsequent VLP formation (12–15). Chimeric VLPs have been used to improve the immune response to defined Ags, like SR1 from *Theileria annulata* (14) or VP1 capsid protein from foot-and-mouth disease virus (15), among others.

The second nanoparticle platform exploits the capacity of spherical hollow silica vesicles (SVs) to adsorb a range of different types and sizes of molecules onto their external and internal surfaces via electrostatic interactions (16). SVs have not been extensively tested as an Ag delivery system because of a lack of uniformity in their synthesis. However, a robust, simple two-step fabrication process of SVs that controls vesicle wall thickness, diameter, and pore size was recently described. The SV surfaces can be functionally modified by amino or hydrophobic groups to empirically optimize the adsorption of Ags (17). SVs with a diameter of 50 nm are of an ideal size for endocytosis and they have been shown to adsorb whole Ags and to enhance the immunogenicity of *Anaplasma marginale* Ags in mice (18) and of the E2 Ag of bovine viral diarrhea virus 1 (BVDV-1) in sheep (19).

In this study, we report on the immunogenicity of p67C following immunization of cattle with s-p67C, chimeric HBcAg VLPs displaying p67C (HBcAg-p67C), s-p67C adsorbed to SV-140-C_18_ SVs (SV-p67C), and a combination of SV-p67C and HBcAg-p67C. All Ags were formulated with the adjuvant ISA 206 VG. In these comparative studies, HBcAg-p67C induced the highest level of p67C Ab responses and a switch in Ab subtype but a poor CD4^+^ T cell response, and SV-p67C induced a strong CD4^+^ T cell response but lower levels of Abs. Immunization with a combination of SV-p67C and HBcAg-p67C induced high p67C-specific Ab and CD4^+^ T cell responses and resulted in the highest levels of protection to *T. parva* sporozoite challenge.

## Materials and Methods

### Bacterial-derived s-p67C, HBcAg-p67C, and control HBcAg VLP production and characterization

To generate s-p67C, residues 572–651 of *T. parva* (Muguga) p67 Ag were cloned as a *Bam*HI-*Hind*III fragment in pET-28a^+^ (Novagen). The total length of the p67C fusion protein is 114 aa residues of which the terminal 80 residues encode p67C (10) (Table I). Bulk production and purification of 100 mg s-p67C was out-sourced to GenScript Biotech. Briefly, *Escherichia coli* pellets were lysed by sonication in guanidine hydrochloride and the target protein obtained by one-step purification using an Ni-affinity column under denaturing conditions. Fractions were pooled, extensively dialyzed against PBS followed by 0.22 μm filter sterilization, and stored at −80°C. As judged by SDS-PAGE, the protein was >95% pure.

To generate HBcAg-p67C, p67C cDNA was amplified from *T. parva* Muguga sporozoite first-strand cDNA prepared using the Omniscript Reverse Transcription Kit (Qiagen). Gene-specific primers (p67C forward: 5′-CCCCgttaacTTGGAAGATGGTGGTGGTGGTTCTGGTGGTGGTGGTGGAACGGGAGGGGGATCA-3′; and p67C reverse: 5′-CCACAATAGCAGCTGGAGGAGAAGGTGGTGGTGGTTCTGGTGGTGGTGGTCCAgctagcCCC-3′) contained a 9-aa linker [(G)_4_S(G)_4_, underlined] and restriction enzyme sites for HpaI or NheI (lowercase) to allow directional cloning of the PCR product first into the pGEM-T easy vector (Promega) and then into pBAD/B-HBcAg (20). Integration of p67C occurs between aa 78 and 79 of HBcAg (Table I). All clones were sequence verified and the three-dimensional structure of the HBcAg-p67C fusion protein was predicted by using the protein structure homology modeling server SWISS-MODEL (21).

HBcAg-p67C and HBcAg protein expression, purification, and formation of VLPs was as previously described (22). These experiments were performed at the Integrated Molecular Plant Physiology Research group of the University of Antwerp. Briefly, a single *E. coli* MC1061 colony containing pBAD/B-HBcAg-p67C was grown overnight in 5 ml of Luria-Bertani (Sigma-Aldrich) broth and used to seed larger cultures before addition of l-arabinose (0.02% w/v; Sigma-Aldrich) for the induction of HBcAg-p67C fusion protein expression. Cells were lysed using a French cell press (10,000 p.s.i.; SLM Aminco), the clarified supernatant was loaded on a 10-ml DEAE Sephacel column (GE Healthcare), and the flow through was collected (25 ml). Following two rounds of ammonium sulfate precipitations, first at 20% and then at 15%, the final pellet was solubilized in 2 ml of wash buffer (50 mM Tris/HCl, 100 mM NaCl, 0.01% Triton-X100, pH8) and dialyzed overnight using 5 l of wash buffer to allow protein refolding and VLP formation. After centrifugation, the supernatant was loaded on a gel filtration column (height: 100 cm, diameter 2.5 cm Sephacryl S-300; GE Healthcare) and protein fractions in the void volume were collected. Control HBcAg VLPs were obtained using the pBAD/B-HBcAg vector, following the same procedure.

Purified proteins were separated by SDS-PAGE, and gels were stained with a Colloidal Blue Staining Kit (Invitrogen) or Coomassie Brilliant Blue (Sigma-Aldrich) and were also analyzed by Western blot with anti-p67C mAb ARIV 21.4 (9) (ascites diluted at 1/1000) and secondary anti-mouse–HRP Ab (Sigma-Aldrich). Protein quantities were measured by a BCA Protein Assay Kit (Pierce) and evaluation of the particle formation was done using electron microscopy on a 2010 Tecnai G2 (FEI) transmission electron microscope. Samples were coated on Formvar/Carbon support film grids (no. FCF200CU; Laboprimex) and negatively stained with 1% phosphotungstic acid (pH 8).

### Identification of different SVs capacity to adsorb s-p67C

A range of different SVs (SV-50, SV-50–NH_2_, SV-50–C_18_, SV-100, SV-100–NH_2_, SV-140, SV-140–NH_2_, and SV-140–C_18_ particles [Table II]) were generated as previously described (23) and were incubated with s-p67C in sterile PBS (Life Technologies). The particle-protein slurry was incubated overnight on a shaker at 200 rpm at 4°C followed by SDS-PAGE analysis of the SVs and supernatant fractions. The relative abundance of Ag adsorbed was ranked arbitrarily depending on the amount of Ag on the supernatants after centrifugation. This led to identification of SV-140–C_18_ SVs as having the highest capacity for adsorption of s-p67C. By varying the ratio of SV to s-p67C, 1 mg of SV-140–C_18_ was found to adsorb 133 μg of s-p67C.

### Synthesis and characterization of SV-140–C_18_ SVs

The two-step production of SVs has been previously described (17). Briefly, in step one, 0.5 g of EO_39_BO_47_EO_39_ [commercial name B50–6600, EO is poly(ethylene oxide) and BO is poly(butylene oxide); Dow Company] and 0.852 g of Na_2_SO_4_ were dissolved in 30 g of pH 4.7 NaAc-HAc buffer solution ([NaAc] = [HAc] = 0.40 M) under vigorous stirring overnight to form a homogeneous solution at 10°C. Then, 3.33 g of tetraethyl orthosilicate (TEOS) was added to the solution with continuous stirring for 24 h at 10°C. In step two, the reaction mixture was placed in an autoclave and hydrothermally treated at 140°C for another 24 h. The precipitate, consisting of SVs, was filtered, repeatedly washed with deionized water to remove the added salts, dried in air, and calcined at 550°C in muffle furnace (Carbolite) in air for 5 h. To modify SVs with octadecyl (-C_18_) groups, 48 mg of calcined SVs were dispersed in 6 ml of toluene (Sigma-Aldrich) in a 50-ml flask. The mixture was stirred at 110°C for 6 h and 0.12 ml of *n*-octadecyltrimethoxysilane (Sigma-Aldrich) was added to the mixture with stirring at 110°C for 12 h. The SVs were recovered by centrifugation, extensively washed with toluene and ethanol, and dried in a fume-hood at room temperature.

The morphology of SV-140–C_18_ particles was observed using a field emission scanning electron microscope (FE-SEM; JEOL JSM 7800) operated at 0.8 kV. A sample-ethanol dispersion was dropped to an aluminum foil piece, dried, and attached to the conductive carbon film on an FE-SEM mount. The FE-SEM mount was then cleaned by the plasma cleaner for 5 min before observation. For transmission electron microscopy observation, the sample-ethanol dispersion was dropped to the carbon film on a Cu grid and dried. Nitrogen adsorption-desorption isotherms were measured by using a Micromeritics Tristar II system at −196°C, before which the sample was degassed at 100°C overnight on a vacuum line. The total pore volume was calculated from the amount adsorbed at a maximum relative pressure (*P/P_0_* = 0.99). The entrance size of SVs was calculated by the Barrett–Joyner–Halanda method from the desorption branch of the isotherms and the specific surface area was calculated from the Brunauer–Emmett–Teller method. Fourier transform infrared (FTIR) spectrum was collected on a ThermoNicolet Nexus 6700 FTIR spectrometer equipped with a Diamond Attenuated Total Reflection Crystal. Thirty-two scans were collected for the spectrum at resolution of 4 cm^−1^ in the range 400–4000 cm^−1^.

Several characteristic peaks were found at 808, 1050–1200, 2852, and 2922 cm^−1^ (Supplemental Fig. 1C), which can be attributed to symmetric –Si-*O*-Si (24, 25) and antisymmetric -CH2- stretching of octadecyl groups (26, 27), respectively.

### SV-p67C–FITC confocal microscopy

An aliquot of s-p67C was labeled with a fluorescent FITC tag using an FITC conjugation kit (Abcam), following the manufacturer’s instructions, hereafter referred to as s-p67C–FITC. SV-140–C_18_ SVs were adsorbed with s-p67C–FITC (SV-p67C–FITC), as described above. Murine macrophage-like RAW 264.7 cells (kindly donated by Dr. B. Rolfe from the Australian Institute for Bioengineering and Nanotechnology, The University of Queensland), were maintained in DMEM supplemented with 10% FBS at 37°C in a 5% CO_2_ incubator using standard cell culture procedures.

For cellular uptake assays, 10^5^ RAW 264.7 cells were allowed to attach on sterile microscope coverslip chambers at 37°C for 24 h and then exposed to SV-140–C_18_ SVs, s-p67C–FITC, and SV-p67C–FITC, the last two formulated with and without the adjuvant ISA 206 VG. Montanide ISA 206 VG adjuvant was mixed with Ag following the manufacturer’s instructions before exposing the mix to the cells. After 2 h of incubation at 37°C and 5% CO_2_, the cells were washed three times with PBS pH 7.4. Acidic organelles in the cells were stained red with Lysotracker Red DND-99 (Thermo Fisher Scientific), cell membrane was stained with wheat germ agglutinin, Alexa Fluor 647 conjugate (WGA-647; Thermo Fisher Scientific) and nuclei were stained with Hoechst 33342 (Thermo Fisher Scientific), following the manufacturer’s instructions. Cells were visualized using a 40× confocal laser-scanning microscope (LSM510METS; Zeiss). Microscopy was performed at the Australian Cancer Research Foundation (ACRF)–Institute for Molecular Bioscience Cancer Biology Imaging Facility, which was established with the support of the ACRF.

### Cattle immunogenicity and challenge studies

Holstein/Friesian and Ayrshire cattle (*Bos taurus*) from 6 to 9 mo old and negative for *T. parva* Abs as determined by ELISA (28) were sourced from farms in the Kenyan highlands. Animal experiments and routine maintenance was in accordance with procedures approved by International Livestock Research Institute’s (ILRI’s) Institute Animal Care and Use Committee (experiment references 2016.15, 2016.24 for immunogenicity studies, and 2017.23 for the challenge experiment).

For the immunogenicity studies, three animals were randomly assigned to one of six experimental groups and each animal received three doses of Ag, with 28-d intervals between booster doses (Table III). Animals received a total of 2 ml per inoculation, administered s.c. in the neck, and were monitored for adverse clinical reactions at the site of inoculation. All immunogens (except for group 4) were diluted in PBS and mixed with Montanide ISA 206 VG adjuvant (Seppic) in a 1:1 ratio following the manufacturer’s instructions. In group 1, animals (BM005, BM062, and BM065) were immunized with 100 μg of purified s-p67C fusion protein (equivalent to 70 μg of p67C); group 2 (BM003, BM015, and BM073) and group 4 animals (BM156, BM163, and BM189) were immunized with 100 μg of s-p67C adsorbed by 752 μg SV-140–C_18_ SVs (SV-p67C) with and without adjuvant ISA 206 VG, respectively; group 3 animals (BM135, BM162, and BM203) were immunized with 300 μg HBcAg-p67 VLPs, equivalent to ∼80 μg p67C. Finally, group 5 (BN046, BN047, and BN048) were immunized with the same amount of SV-67C and HBcAg-p67C (from now called SV-HBc-p67C and equivalent to ∼150 μg of p67C) separately formulated with ISA 206 VG and given as two inoculations, one of each side of the neck; and group 6 (BN096, BN101, and BN120) were inoculated with SV-HBc-p67C formulated with ISA206 VG together in a single inoculation. None of the animals exhibited immediate or delayed hypersensitivity, indicating safety of the Ag formulations. Samples of blood for preparation of serum and PBMCs were taken at various times as indicated in the text.

For the challenge experiment, 30 cattle were allocated in two groups of 15 each (Table III). Group 7 animals received three doses (with 28-d intervals between booster doses) of SV-HBc-p67C (equivalent to ∼150 μg of p67C), separately formulated with ISA 206 VG and given as two inoculations, one of each side of the neck. Group 8 animals were kept unvaccinated to be used as a control group for challenge. A group of animals vaccinated with HBcAg alone was not included, as it was previously reported it has no effect on challenge outcome (29, 30). Twenty-one days after the last boost, all animals were given an s.c. syringe challenge of 1 ml of *T. parva* Muguga sporozoites (stabilate no. 3087), as previously described (11). After the challenge, all experimental cattle were monitored daily for changes in rectal temperatures and other clinical manifestations of ECF, and the ECF scores were calculated (Rowland index) (31). The index was used to define whether animals were susceptible (index: 6–10, nonprotected) or immune (index: 0–5.99, protected) to ECF by the end of the experiment, as previously described (7).

### Statistical analysis for cattle in vivo experiments

The differences among immunogenicity groups in response to p67C was also analyzed using a Kruskal–Wallis rank sum test, a nonparametric method for testing whether samples originate from the same distribution.

Analysis of the immunity status of challenged animals was carried out using both nonparametric and parametric methods. The immune animals, as defined by the ECF scores described above, were first coded 1 and nonimmune animals were coded 0. The nonparametric difference in proportion of animals immune between group 7 and group 8 was evaluated using a Fisher exact test and exact 95% confidence intervals were calculated. Subsequently, a parametric logistic regression was used to test the effect of group, breed, and age on probability of immunity. As breed and age were nonsignificant, the model was refit containing group only.

Analysis of the ECF score was also carried out using parametric methods. Prior to statistical analysis of the final ECF scores, animals who reached the humane end point (ECF score of 6.5) and were removed from the trial prior to the end of the experiment (21 d after challenge) had their final ECF score fixed at 6.5. Linear regression analysis was used to test for the differences in ECF scores with group, breed, and age as explanatory variables. As with immunity, the breed and age were nonsignificant. The regression was rerun containing only the group effect and least-square mean final ECF score and their 95% confidence intervals calculated for each group. The residuals were checked to confirm the assumptions of the linear regression were appropriate, despite the fixed nature of animals who passed the humane end point (6.5) these assumptions (linearity, normality, homoscedasticity, lack of multicollinearity, and autocorrelation) were reasonable.

A Fisher exact test to assess the association between immunity and a binary variable half-maximal (half-max) Ab titers and CD4 indices was also performed to be able to assess the relationship of these two parameters, separately and in combination, with protection. The half-max titer of 6000 and a CD4 index of 100 were selected as a cut-off for the association because they are approximately the mean response of the immune animals in group 7. For the combination variable, 1 = both half-max Ab titers and CD4 indices above threshold versus 0 = all other combinations. A 95% exact confidence interval was calculated around the observed immunity percentages for the above threshold half-max Ab titers, CD4 titers, and their combination.

### ELISAs for detection of Ab isotypes and sporozoite seroneutralization assay

p67C-specific IgG and IgM Abs isotypes and IgG1 and IgG2 subtypes responses were detected as previously described (11). Briefly, plates were coated with 0.5 μg/ml of Ag in PBS for an ELISA and developed with sheep anti-bovine IgG, IgM, IgG1, or IgG2 HRP-conjugated secondary Abs (Bio-Rad Laboratories). Half-max Ab titers were calculated using OD values from an Ab dilution series with Graph Pad Prism version 7.0b (GraphPad software). HBcAg-specific Abs were detected in a similar assay using HBcAg particles at 5 μg/ml in PBS as the coating Ag.

The ability of heat-inactivated bovine sera to inhibit in vitro infectivity of the *T. parva* sporozoites was assessed using a 96-well seroneutralization assay as previously described (11). Briefly, 1/50 dilutions of heat-inactivated sera were incubated with *T. parva* sporozoites for 10 min. Fresh Ficoll-isolated cattle PBMC were added to the mix and incubated for 1 h. Thereafter, plates were washed and resuspended in RPMI 1640 supplemented with 10% heat-inactivated FBS and incubated for 14 d at 37°C. The read-out of the assay was based on an anti-PIM cellular ELISA, a parasite Ag that is expressed by the schizont stage of the parasite. Sporozoite neutralization results were expressed as the percentage of reduction of infectivity compared with infectivity rates in the presence of preimmunization sera (day 0).

### Serological specificity to overlapping p67C synthetic linear peptides

Nine 15-mer peptides overlapping by 7 aa (Mimotopes Pty) were designed to assess the peptide epitope specificity of sera from immunized animals (Supplemental Table I). Immobilizer amino ELISA plates (Nunc Cell Culture) were coated overnight at 4°C with 10 μg/ml (100 μl per well) of each peptide in 100 mM carbonate buffer (pH 9.6) (Sigma-Aldrich). After incubation, plates were blocked for 1 h at 37°C with PBS containing 0.05% Tween 20 and 2% BSA (Sigma-Aldrich). After blocking, individual bovine sera were added at 1/1000 dilution in blocking buffer and incubated at 37°C for 2 h. Thereafter, bovine Abs were detected using an HRP-conjugated sheep anti-bovine IgG (AAI23P; Bio-Rad Laboratories) diluted 1/5000 in blocking buffer. After 1 h of incubation, the reaction was developed for 10 min at 37°C with 50 μl of TMB plus 2 (Kem-En-Tec Diagnostics) and stopped using 50 μl of 0.5 M H_2_SO_4_ (Sigma-Aldrich). Reactions were read at 450 nm using a Synergy HT ELISA Reader (BioTek Instruments). Plates were washed four times in between each step using PBS containing 0.05% Tween 20 and assays were carried out in triplicates. The results presented as a heat map for the standardized OD from individual animals with the average of the triplicates from each animal and peptide was generated using the Heatmapper software (32). The standardization was performed using the OD against a peptide pool containing all linear peptides as a positive control.

### CD4^+^ T cell IFN-γ ELISpot and ^3^H-thymidine proliferation assays

Assays were carried with CD4^+^ T cells that were enriched from Ficoll-isolated PBMCs using magnetic beads (Miltenyi Biotec) coated with anti-bovine CD4 ascites (ILRI mouse hybridoma clone ILA11), as previously described (11).

IFN-γ ELISpot assay on CD4^+^ T cells was as previously described (33). Briefly, a monoclonal anti-bovine IFN-γ Ab (MCA1783; Serotec) was incubated overnight at 4°C on ELISpot plates (MilliporeSigma), and then blocked with RPMI 1640 supplemented with 10% FBS for 2 h at 37°C prior to adding specific Ag. Ags were tested in triplicates and two different cell concentrations: 1.25 × 10^5^ and 2.5 × 10^5^ cell per well. The plates were incubated at 37°C for 20 h and developed using a rabbit polyclonal anti-bovine IFN-γ Ab (A2556; Sigma-Aldrich). Results were expressed as IFN-γ–secreting cell counts per million CD4^+^ cells (IFNγ-SC per million CD4^+^ cells). Two types of antigenic stimuli were used: s-p67C at 20 μg/ml (equivalent to 2.5 μM) or a pool of seven 25-mer synthetic p67C peptides at 2 μM (Supplemental Table I) that overlap by 16 aa residues covering the p67C sequence (Mimotopes Pty). Media were used as a general negative control, and 25-mer peptides not related to *T. parva* (Mimotopes Pty.) and OVA (Sigma-Aldrich) were used as negative controls. Con A at 2.5 μg/ml was used as a positive control.

CD4^+^ T cell proliferation to different Ags was analyzed in triplicates by means of a ^3^H-thymidine CD4^+^ T cell proliferation assay, as previously described (11). Briefly, CD4^+^ T cells (2 × 10^5^ cells per well) were incubated for 4 d at 37°C in a CO_2_ incubator with the stimulus. Four stimuli were used in this assay: s-p67C at 20 μg/ml (equivalent to 2.5 μM); a pool of seven 25-mer synthetic p67C peptides at 2 μM (Supplemental Table I) overlapping in 16 aa; and SV-p67C and HBcAg-p67C, each adjusted to an equivalent amounts. Moreover, in this assay, two extra negative controls were also included, apart from media alone, not–*T. parva* related peptides, and OVA: SV-140–C_18_ empty SVs and HBcAg at 55 μg/ml (equivalent amount of core Ag than into HBcAg-p67C). ConA at 2.5 μg/ml was used as a positive control. The results were expressed as fold-increase resulting from CPM-counts^stimuli^/CPM-counts^media^. Cells incubated with media never had CPM values under 1022. As assays were carried out at different times the fold-increase indices were standardized dividing the original index by the ConA index (positive control) and multiplied by the average of ConA indices from the different animals in different assays.

## Results

### HBcAg-p67C and control HBcAg VLP Ag production and characterization

**FIGURE 1.**
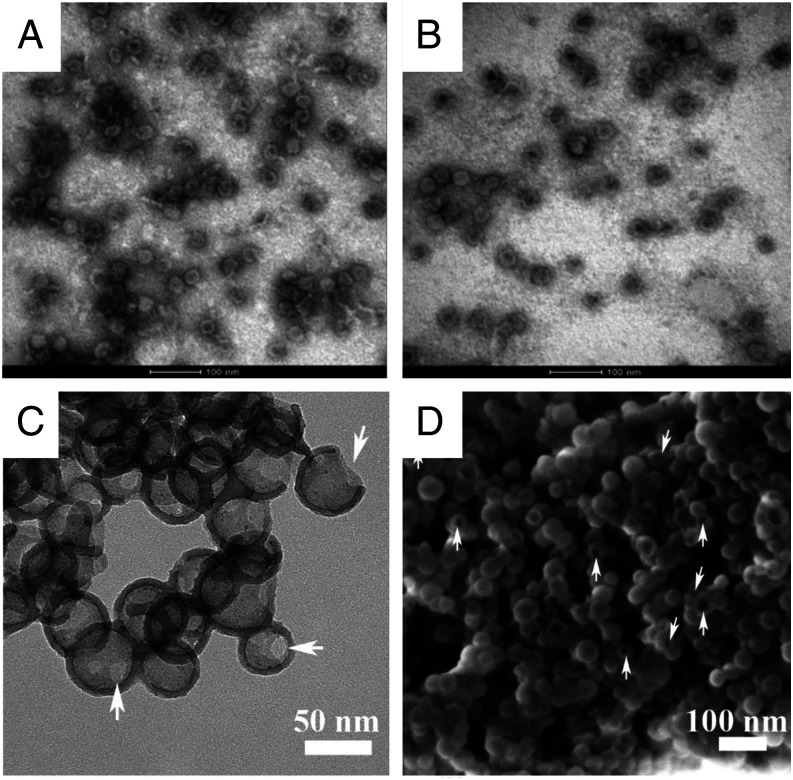
Electron microscope (EM) images of VLPs and SV-140–C_18_. Transmission EM analysis of (**A**) HBcAg-p67C VLPs, (**B**) HBcAg VLPs, and (**C**) SV-140–C_18_, as well as (**D**) scanning EM analysis of SV-140–C_18_. Arrows indicate entrances on the shells of SVs in both (C) and (D).

HBcAg-p67C and control HBcAg VLPs were purified under nondenaturing conditions from bacterial cell lysates, folded into VLPs and assessed using electron microscopy. Particles around 30–35 nm with icosahedral-like symmetry were observed (Fig. 1A, 1B). An mAb, ARIV21.4, which binds to an epitope on p67C (9) recognized s-p67C and HBcAg-p67C but not HBcAg on Western blots (data not shown). Average yields of ∼8.5 mg of s-p67C, ∼11 mg of HBcAg-p67C VLPs, and ∼13 mg of HBcAg VLPs were obtained from 1 L of *E. coli* cultures (Table I).

**Table I.** Amino acid sequence of proteins used in the experiments

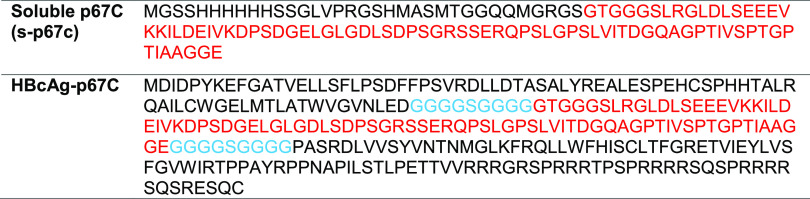

Amino acid residues in red mark p67C sequences, and those in blue mark a linker sequence.

### Production and characterization of SV-140–C_18_ SVs and SV-p67C

To identify which type of SV particles efficiently adsorb s-p67C, SVs with different diameters and surface chemistries were mixed overnight at 4°C with s-p67C in sterile PBS followed by p67C-adsorption analysis of the SVs and supernatant. The s-p67C was shown to adsorb to many of the SVs (Table II). The SV-140–C_18_ particles, which are 50 nm in diameter and surface modified with octadecyl groups (-C_18_), were found to have the highest adsorption capacity for s-p67C, with 1 mg of particles adsorbing 133 μg of s-p67C.

**Table II.** Summary of SV properties and assessment for p67C adsorption

SV Names	Size (nm)	Surface Charge	Properties	Entrance Pore Size	Relative Abundance of Unbound s-p67C
SV-50	47.6 ± 2.2	Negative	Hydrophilic	Small	++
SV-50–NH_2_	49.9 ± 2.7	Positive	Hydrophilic	Small	+++
SV-50–C_18_	50.7 ± 3.1	Slightly negative	Hydrophobic	Small	+++
SV-100	47.9 ± 5.0	Negative	Hydrophilic	Medium	++
SV-100–NH_2_	49.1 ± 1.9	Positive	Hydrophilic	Medium	++
SV-140	51.7 ± 3.5	Negative	Hydrophilic	Large	++
SV-140–NH_2_	51.1 ± 1.4	Positive	Hydrophilic	Large	+++
SV-140-C_18_	52.4 ± 5.1	Slightly negative	Hydrophobic	Large	+

SV-140–C_18_ were fabricated and characterized as previously described (34). Electron microscope analyses revealed uniform spheres with pores (Fig. 1C, 1D). The average particle diameter and shell thickness were calculated to be 52.4 nm ± 5.1 nm and 5.7 nm ± 0.4 nm, respectively, with a pore size of 14.9 nm (Supplemental Fig. 1A). The total pore volume and Brunauer–Emmett–Teller surface area of the SV-140–C_18_ were 0.575 cm^3^g^−1^ and 131 m^2^g^−1^, respectively (Supplemental Fig. 1B). The FTIR spectrum of SV-140–C_18_ indicated the presence of octadecyl group modifications on the silica surfaces (Supplemental Fig. 1C). A typical round of synthesis yielded ∼800 mg of SV-140–C_18_ particles.

### SV-p67C–FITC–labeled vesicles can access an acidic intracellular compartment

**FIGURE 2.**
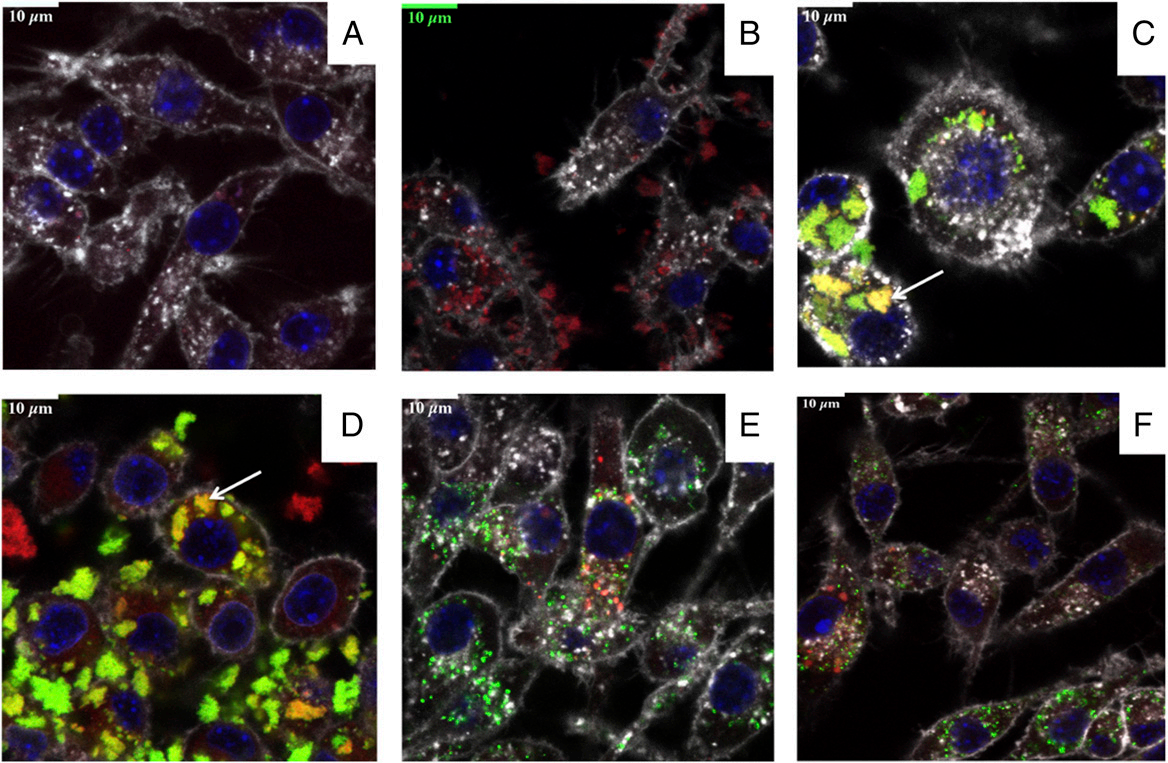
Confocal fluorescence microscopy of RAW 264.7 cells following incubation with SVs. The images are of (**A**) RAW 264.7 cells alone, (**B**) cells with SV-140–C_18_, (**C**) cells with SV-p67C–FITC, (**D**) cells with SV-p67C–FITC in the presence of ISA 206 VG, (**E**) s-p67C–FITC, and (**F**) s-p67C–FITC in the presence of ISA 206 VG. Acidic organelles in the cells were stained red with Lysotracker Red DND-99, cell membranes were stained white with wheat germ agglutinin, Alexa Fluor 647 conjugate (WGA-647) and nuclei were stained blue with Hoechst 33342. FITC is represented in green, and the colocalization of Ag in endosomes appears orange because of an overlap of red and green (white arrows indicate the colocalization of Ag in the endosomes). The assays were performed in duplicate, and the best images are shown.

To infer a possible intracellular fate of SV-p67C in vivo, we used confocal microscopy to evaluate the in vitro interaction of a mouse macrophage cell line, RAW 264.7, with s-p67C labeled with FITC adsorbed to SV-140–C_18_ and costained with cellular markers. No autofluorescence was observed in RAW 264.7 cells alone (Fig. 2A) or in cells incubated with SV-140–C_18_ (Fig. 2B) as only the endosomes (red), cell membrane (white), and the nuclei (blue) were stained. Cells incubated with SV-p67C–FITC alone (Fig. 2C) and following formulation with ISA 206 VG (Fig. 2D) resulted in colocalization of Ag in an acidic compartment (orange and arrows in Fig. 2C, 2D), which is likely to be part of the endosomal system. This phenomenon was not observed either when incubating the cells with s-p67C–FITC with or without ISA 206 VG.

### High p67C-specific Ab responses were stimulated by HBcAg-p67C, whereas SV-p67C stimulated higher CD4^+^ T cell responses in immunogenicity studies

We next carried out a series of immunogenicity studies with p67C in the three different Ag formulations. Three Holstein/Friesian cattle were assigned per experimental group (Table III), and group 1, 2, and 3 animals were inoculated with s-p67C, SV-p67C, and HBcAg-p67C, respectively, with each animal receiving an equivalent of 70–80 μg of p67C Ag per inoculation mixed with ISA 206 VG adjuvant. Group 4 animals received SV-p67C without adjuvant.

**Table III.** Summary of animal groups for immunogenicity studies (groups 1–6) and challenge experiments (groups 7 and 8)

Group	Immunogen	Doses	Adjuvant	p67C (μg) per Inoculation	Total Protein (μg) per Inoculation	Number of Animals	Challenge
Group 1	s-p67C	3	ISA 206 VG	∼70–80	100	3	N/A
Group 2	SV-p67C	3	ISA 206 VG	∼70–80	100	3	N/A
Group 3	HBcAg-p67C	3	ISA 206 VG	∼70–80	300	3	N/A
Group 4	SV-p67C	3	N/A	∼70–80	100	3	N/A
Group 5	SV-HBc-p67C (two inoculations)	3	ISA 206 VG	∼140–150	400	3	N/A
Group 6	SV-HBc-p67C (one inoculation)	3	ISA 206 VG	∼140–150	400	3	N/A
Group 7	SV-HBc-p67C (two inoculations)	3	ISA 206 VG	∼140–150	400	15	LD_93_
Group 8 (challenge control)	N/A	N/A	N/A	N/A	N/A	15	LD_93_

HBcAg-p67C, chimeric HBcAg VLPs displaying p67C; N/A, not applicable; SV-HBc-p67C, SV-p67C + HBcAg-p67C.

**FIGURE 3.**
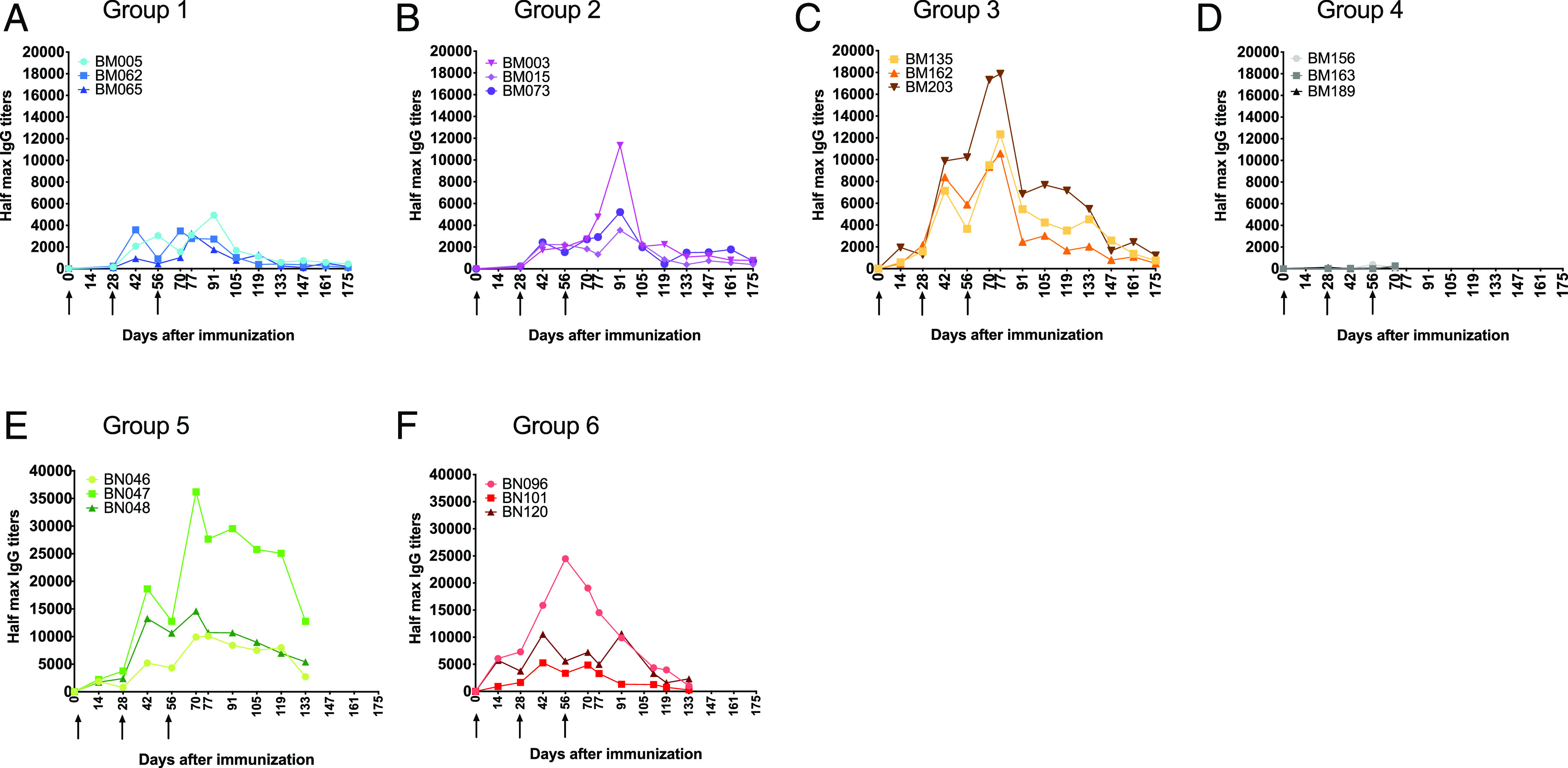
Half-max p67C-specific total IgG Ab titers present in sera in cattle from the immunogenicity studies (three animals per group). Ab titers were measured at different time points in individual animals in groups 1–6: (**A**) s-p67C, (**B**) SV-p67C, (**C**) HBcAg-p67C, (**D**) SV-p67C without ISA 206 VG adjuvant, (**E**) SV-p67C + HBcAg-p67C as two separate inoculations, and (**F**) SV-p67C + HBcAg-p67C as one inoculation. The black arrows mark the day of Ag inoculation. Note the difference in the *y*-axis scale for (A)–(D) versus (E) and (F).

Animals in group 3 developed an earlier and higher IgG Ab titers to p67C than those in groups 1 and 2 (Fig. 3A–C). Although individual animal variation in Ab levels were observed in all groups, there was a clear, positive effect associated with p67C inoculated as VLPs (Fig. 3C). The average IgG half-max titers at day 77 in group 3 was approximately five times higher than the average titers in groups 1 and 2 (Table IV). Although SVs provided an adjuvant effect in sheep (35), in our studies, inoculation of SV-p67C without ISA 206 VG gave a very poor Ab response (Fig. 3D), and the group was not followed beyond day 70. No significant levels of IgM were detected at any time point to day 77 in any of the treatment groups.

**Table IV.** Summary of ELISA results for immunogenicity studies in cattle

Group	Animal ID	p67C-Specific IgG Half-Max Ab Titers		Ratio IgG1/IgG2 (Day 77)	p67C-Specific IgG1 Half-Max Titers	p67C-Specific IgG2 Half-Max Titers
		Day 77	Day 91			
Group 1 (s-p67C)	BM005	3,127	4,943	3,207	3,207	Not detectable
	BM062	2,792	2,734	2,600	2,600	Not detectable
	BM065	3,256	1,761	12	3,150	255
Group 2 (SV-p67C)	BM003	4,757	11,342	5,000	5,000	Not detectable
	BM015	1,329	3,546	1,246	1,246	Not detectable
	BM073	2,921	5,216	3,100	3,100	Not detectable
Group 3 (HBcAg-p67C)	BM135	12,337	5,461	7	12,274	1,682
	BM162	10,568	2,460	15	11,345	753
	BM203	17,883	6,858	8	16,394	2018
Group 4 (SV-p67C without ISA 206 VG)	BM156	76*^a^*	N/A	N/A	N/A	N/A
	BM163	265*^a^*	N/A	N/A	N/A	N/A
	BM189	105*^a^*	N/A	N/A	N/A	N/A
Group 5 (SV-HBc-p67C, two inoculations)	BN046	10,087	8,410	13	11,200	862
	BN047	27,652	29,544	3	25,400	8,830
	BN048	10,715	10,671	3	9,987	3,782
Group 6 (SV-HBc-p67C, one inoculation)	BN096	14,523	9,884	11.87	8,895	750
	BN101	3,299	1,317	18.12	3,800	210
	BN120	4,983	10,641	4.39	6,354	1,447

^a^ Day 70 p67C-specific IgG half-max Ab titers.

ID, identifier; N/A, not applicable.

The longevity of the Ab response was monitored in groups 1–3 for several weeks after the last Ag boost (Fig. 3). A multiphasic decay of Abs was present in group 3, in which there was a sharp fall in Ab levels after a peak at day 77, which was followed by stabilization of Ab levels from day 91 to 119 and then another fall in Ab titer after day 133. The Ab levels remained relatively stable from then to the end of the experiment (day 175) (Fig. 3C). Increase in Ab titers following each Ag dose were more modest in groups 1 and 2 and only observed after the second Ag boost in group 2 but not until day 91 (Fig. 3B). Unexpectedly, sera from group 3 animals contained very low levels of Abs to HBcAg VLPs (Supplemental Fig. 2).

**FIGURE 4.**
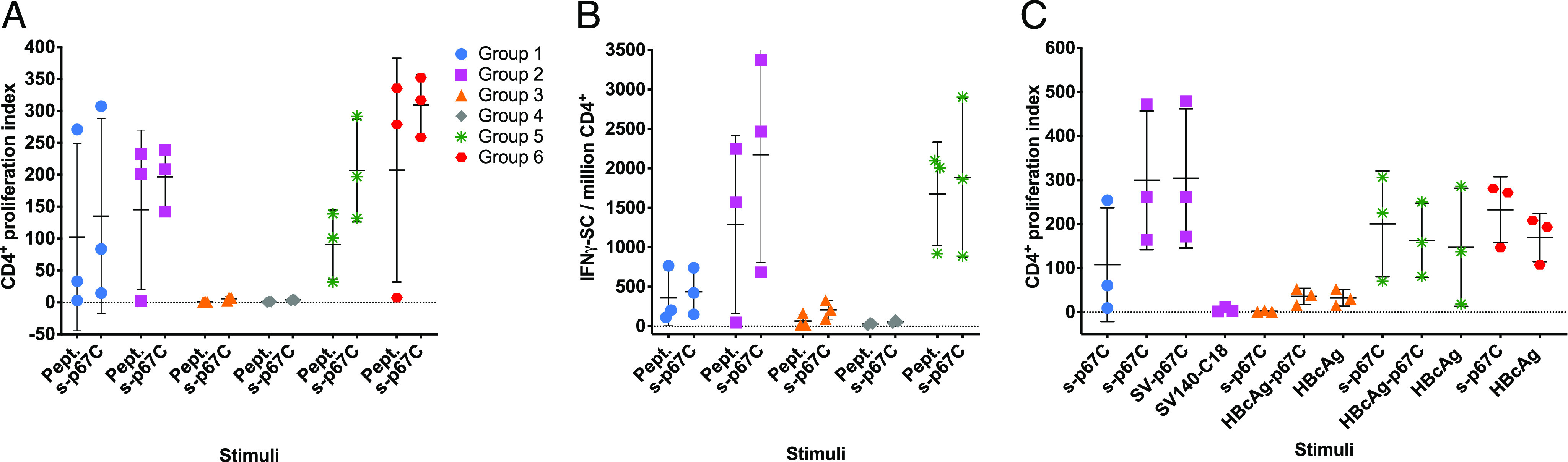
The responses of enriched CD4^+^ T cell from immunized cattle to different stimuli were measured in cattle from the immunogenicity studies (three animals per group). (**A**) The p67C-specific proliferative cellular response in individual animals in groups 1–6 (Kruskal–Wallis *p* < 0.05) and (**B**) p67C-specific IFN-γ–secreting cells per million CD4^+^ T cells in individual animals in groups 1–4 were measured at 2 wk after the last boost at day 70 (Kruskal–Wallis *p* < 0.05). (**C**) T cell proliferative responses were measured in individual animals in groups 1–3 and groups 5 and 6 at day 175 to different stimuli. Ag stimuli used were the following: p67C peptide pool (a pool of 25-mer p67C–overlapping peptides), s-p67C, SV-p67C, SV-140–C_18_ SVs, HBcAg-p67C, and HBcAg VLPs. The group average and SD are also shown.

To measure T cell responses to p67C, MACS bead–purified CD4^+^ T cells from cattle were stimulated with s-p67C protein or a pool of 25-mer p67C–overlapping synthetic peptides (Supplemental Table I). A CD4^+^ T cell proliferation (Fig. 4A) and an IFN-γ ELISpot assay (Fig. 4B) were conducted at day 70. In both assays, the strongest responses were observed in group 2, followed by group 1, with groups 3 and 4 giving the lowest responses (Table V). To investigate the longevity of T cell responses, the CD4^+^ proliferation assay was repeated with samples collected at day 175, ∼4 mo, after the final Ag dose (Fig. 4C). As before, animals from group 2 gave the highest response, but they did not proliferate in response to SV-140–C_18_, indicating that the SVs do not provide a nonspecific antigenic stimulus in vitro. Cells from animals in group 3 proliferated mainly to HBcAg, indicating that T cell responses were directed to hepatitis B core Ag sequences rather than p67C in this Ag format. Chimeric HBcAg VLPs have also been reported to prime Ag-specific CD8^+^ T cell responses (36). However, we were unable to measure a significant IFN-γ ELISpot response to overlapping p67C synthetic 25-mer peptides in an assay using purified CD8^+^ T cells from cattle in groups 1, 2, or 3 (data not shown).

**Table V.** Summary of CD4^+^ proliferation and IFN-γ results for immunogenicity studies in cattle

Group	Animal ID	T Cell CD4^+^ Proliferation Index*^a^*	IFN-γ Spots per Million CD4^+^ T Cells*^a^*
Group 1 (s-p67C)	BM005	83.66	422.42
	BM062	307.41	739.84
	BM065	14.51	150.10
Group 2 (SV-p67C)	BM003	208.71	3372.13
	BM015	142.37	683.80
	BM073	238.92	2467.90
Group 3 (HBcAg-p67C)	BM135	7.31	328.91
	BM162	7.64	205.93
	BM203	2.54	92.14
Group 4 (SV-p67C without ISA 206 VG)	BM156	4.8	80.98
	BM163	3.6	40.01
	BM189	2.8	51.72
Group 5 (SV-HBc-p67C, two inoculations)	BN046	197.15	887.78
	BN047	291.45	1861.57
	BN048	131.62	2901.23
Group 6 (SV-HBc-p67C, one inoculation)	BN096	352.28	N/A
	BN101	316.87	N/A
	BN120	258.66	N/A

^a^ Using p67C protein at 2.5 μM as stimuli at day 70.

ID, identifier; N/A, not applicable.

### Simultaneous inoculation of SV-p67C and HBcAg-p67C stimulated high p67C Ab and CD4^+^ T cell responses

To investigate if the HBcAg-p67C and SV-p67C formulations would stimulate both arms of the immune response, we inoculated two groups of cattle with these nanoparticles, delivered in the presence of adjuvant as two simultaneous inoculations (group 5) or as a combined single adjuvant formulation in one inoculation (group 6 and Table III). The animals in groups 5 and 6 received an equivalent of 140–150 μg of p67C Ag per inoculation, double the dose of p67C given to groups 1–4. The first inoculation strategy primed a strong p67C Ag-specific Ab response (Fig. 3E), which was, on average, double the response observed in group 6 (Fig. 3F, Table IV). Relative to the Ab levels in group 5, the levels in group 6 dropped rapidly and were very low by day 133 (Fig. 3F). Interestingly, the decay of Ab levels in group 5 was much slower than that seen in group 3 (Fig. 3C, 3E), and in contrast to group 3 cattle, two of the three animals in group 5 and all group 6 animals developed a strong Ab response to HBcAg VLPs, indicating that cattle can mount an Ab response to the viral Ag (Supplemental Fig. 2). Both group 5 and group 6 cattle developed high levels of p67C-specific CD4^+^ proliferative T cell responses (Fig. 4A). These cattle also developed a T cell responses to HBcAg that were much higher than those seen in group 3 (Fig. 4C). These results suggest that coformulation of HBcAg-p67C with SV-p67C disrupts Ab responses to the carrier proteins on the VLPs and that SVs may stimulate T cell responses per se.

### Anti-p67C Ab subtype responses and specificity to p67C synthetic peptides

**FIGURE 5.**
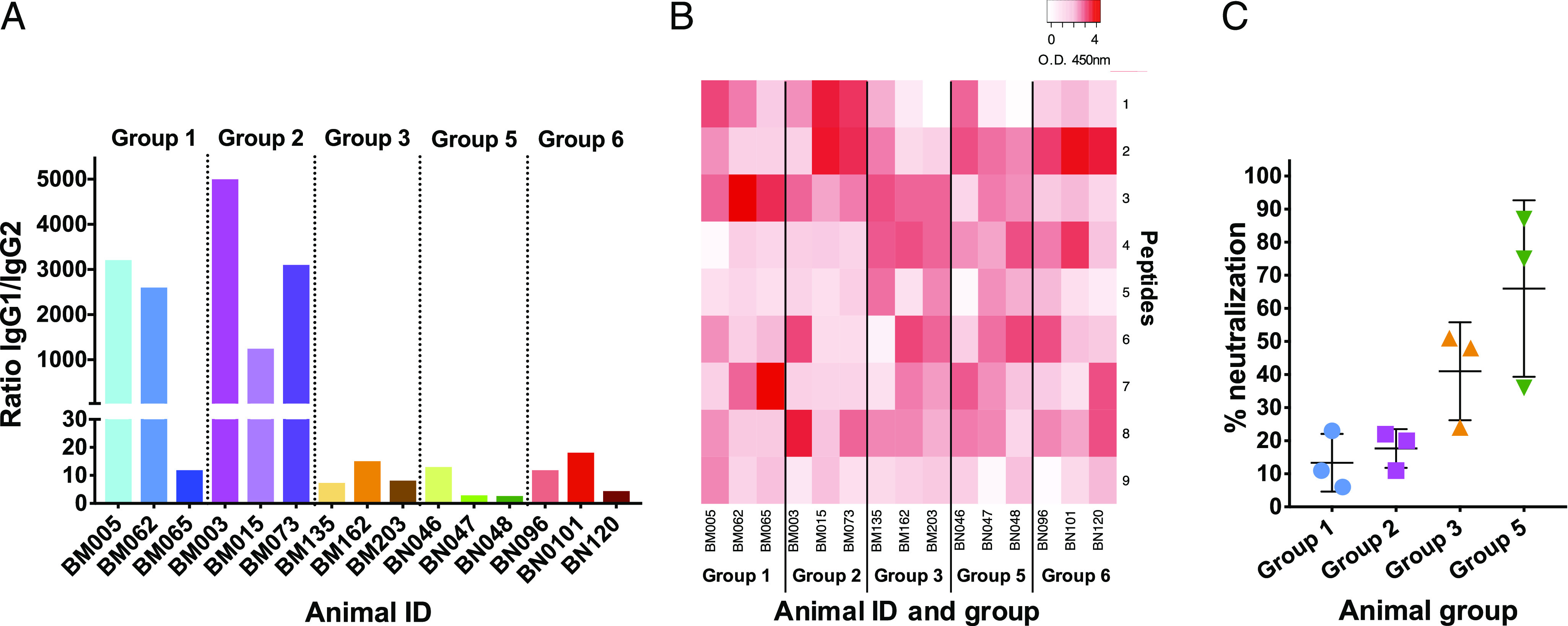
Anti-p67C Ab subtype responses, specificity to p67C synthetic peptides, and sporozoite-neutralizing activity in immunogenicity studies (three animals per group). (**A**) Serum samples from cattle at day 77 after the first Ag dose were used to measure IgG subtype responses, and the p67C-specific Ab IgG1/IgG2 ratios in sera from individual animal in groups 1–3 and 5–6 are shown. (**B**) Reactivity of the same sera with synthetic overlapping p67C peptides using a color-coded heat map generated with Heatmapper software based on peptide reactivity. (**C**) Sporozoite neutralization capacity of serum from individual animals and the group average and SD of sera from groups 1, 2, 3, and 5.

To assess Ab switching, we measured IgG1 and IgG2 ELISA half-max titers to p67C at day 77 in cattle groups 1–6, except group 4 as very poor Ab responses were detected in these animals (Fig. 5A). IgG1 is the predominant IgG subtype expressed in serum and milk in cattle (37), and it was the dominant subtype response detected in the experimental groups analyzed. An IgG2 response was only detected in group 3, 5, and 6 cattle, which received HBcAg-p67C Ag and in one animal in group 1 (Table IV). Thus, cattle in groups 1 and 2 exhibited a skewed IgG1/IgG2 ratio, relative to those in groups 3, 5, and 6 (Fig. 5A).

Using the same sera, we also mapped the binding of total IgG Ab in an ELISA to nine 15-mer synthetic peptides of p67C and were overlapped by 7 aa residues (Supplemental Table I). An Ab response to the peptides was detected with all sera, but the pattern and strength of peptide recognition differed between animals, even within the same group (Fig. 5B). Peptide 6 and peptide 7 contain the sequence SERQPSL and PSLVITD, respectively, which have previously been identified as sites recognized by sporozoite-neutralizing mAbs (9). Ab binding to these peptides by the cattle sera was also variable. Interestingly, the pattern of peptides recognized by group 5 or 6 sera did not represent a simple sum of the specificities present in groups 2 and 3 (Fig. 5B).

### Induction of sporozoite-neutralizing activity

Heat-inactivated day-77 sera diluted 1/50 from group 1, 2, 3, and 5 cattle were tested for their capacity to neutralize *T. parva* sporozoite infectivity in vitro. Group 6 animals were not included in this analysis because delivering the two nanoparticles in one inoculum was not beneficial for immunity compared with group 5. All sera had some degree of neutralizing activity (Fig. 5C, Table VI) with the highest activity (from 48 to 87%) in sera from animals in group 3 (BM135 and BM203) and group 5 (BN047 and BN048). Preimmunization sera from day 0 had no neutralizing capacity. The neutralizing capacity of the sera from group 2 was similar to the animals in group 1 (Fig. 5C). We also measured the sporozoite-neutralizing capacity of sera from group 2 animals at day 91, corresponding to the delayed Ab peak, but this was no higher than that at day 77 (Table VI). Sporozoite-neutralizing activity was found in group 3 and group 5 sera as early as day 14, but only at day 28 in groups 1 and 2 (Table VI).

**Table VI.** Summary results of the percentage of neutralization capacity of sera from animals under immunogenicity studies in cattle

Group	Animal ID	Day 14	Day 28	Day 77	Day 91
Group 1 (s-p67C)	BM005	0	32	23	17
	BM062	0	20	11	6
	BM065	0	9	6	3
Group 2 (SV-p67C)	BM003	0	22	11	13
	BM015	0	19	20	10
	BM073	0	9	22	10
Group 3 (HBcAg-p67C)	BM135	23	47	48	40
	BM162	23	13	24	19
	BM203	20	13	51	60
Group 5 (SV-HBc-p67C, two inoculations)	BN046	8	2	36	14
	BN047	12	28	87	81
	BN048	26	30	75	54

ID, identifier.

### Protection to ECF in cattle inoculated with SV-p67C and HBcAg-p67C as separate formulations

**FIGURE 6.**
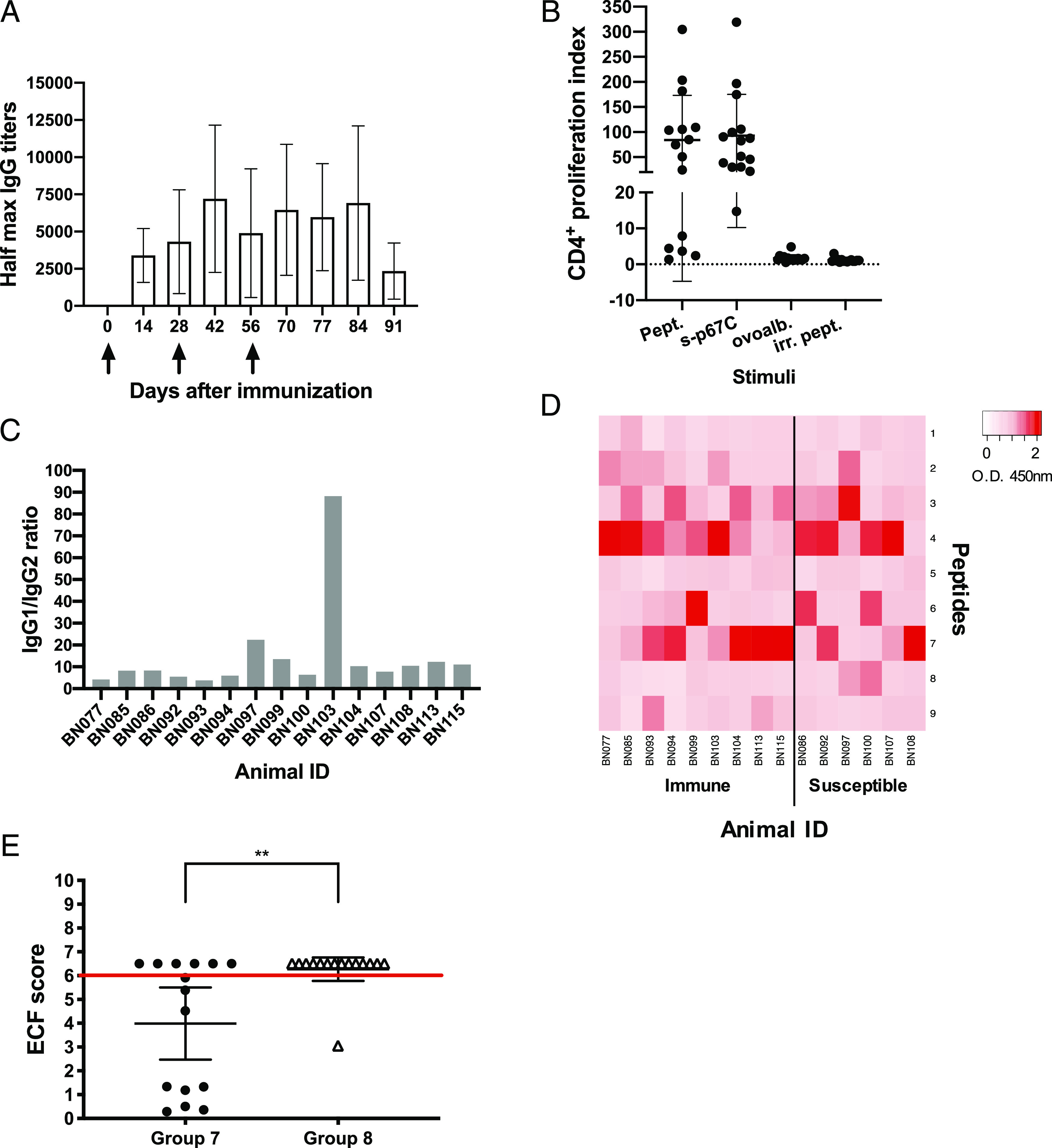
Immune response and protection achieved in group 7 (three doses of HBcAg-p67C/SV-p67C) and group 8 (challenge control) animals, 15 animals per group. (**A**) Kinetics of Ag-specific IgG Ab titers measured in sera, average (*n* = 15), and SD are shown. The days of Ag injection are represented by black arrows. (**B**) p67C-specific CD4^+^ T cell proliferation increases were measured at 2 wk after the last boost (day 70), individual animals (*n* = 15), the group average, and SD are shown. Two different stimuli were used: a pool of 25-mer p67C–overlapping peptides (Pept.) and s-p67C. Negative stimulus responses are also included: ovalbumin (ovoalb.) and irrelevant peptides (irr. pept.). (**C**) p67C-specific Ab IgG1/IgG2 ratios in sera from individual animals. (**D**) A color-coded heat map based on peptide recognition by sera from individual animals is shown. Animals are separated in immune and susceptible groups after challenge. (A–D) Group 7 results. (**E**) ECF scores of both groups of animals, the mean and the 95% confidence interval are shown. A red line separates the protected and nonprotected animals (ECF score ≥ 6). The significance of the differences among groups is also shown. **p < 0.01.

Because only group 5 cattle had high Ab levels with a switch to IgG2 and strong CD4^+^ T cell responses to p67C, we designed an experiment to test the vaccine efficacy of the combination of the two delivery systems, SV-p67C and HBcAg-p67C, from now called “SV-HBc-p67C.” Fifteen Holstein/Friesian cattle (group 7) were immunized as described for group 5 (Table III). The total IgG Ab response (Fig. 6A), CD4^+^ proliferative T cell responses (Fig. 6B), IgG1 and IgG2 subtypes (Fig. 6C), and Ab specificity to synthetic p67C peptides (Fig. 6D) were measured in the immunized cattle (Table VII). All cattle developed IgG and T cell responses to p67C and an Ab response to HBcAg VLPs (Supplemental Fig. 2). However, on average, the total IgG levels to p67C across group 7 cattle were lower than those measured in the group 5 animals (Fig. 3E), and except for one animal, both IgG1 and IgG2 subtypes were present and very balanced in all animals (Fig. 6C, Table VII).

**Table VII.** Summary of immune parameters measured in group 7 (SV-HBc-p67C)

Animal ID	p67C-Specific IgG Half-Max Ab Titers	Ratio IgG1/IgG2 (Day 77)	T Cell CD4^+^ Proliferation Index*^a^*
BN077	13,700	4.21	105.84
BN085	3,033	8.17	5.86
BN086	5,579	8.25	196.68
BN092	1,823	5.45	29.9
BN093	2,590	3.83	51.59
BN094	2,747	5.99	14.74
BN097	2,443	22.40	21.61
BN099	2,776	13.53	38.62
BN100	8,575	6.36	82.75
BN103	6,281	88.24	90.61
BN104	11,344	10.31	99.37
BN107	5,928	7.81	30.27
BN108	6,030	10.49	87.65
BN113	9,752	12.28	174.75
BN115	6,922	11.03	319.37

^a^ Using p67C protein at 2.5 μM as stimuli at day 70.

ID, identifier.

Group 7 cattle were given a needle sporozoite challenge 21 d after the last Ag boost. Fifteen naive cattle (group 8) served as a sporozoite challenge control (Table III). Animals were clinically monitored and an ECF index calculated from the onset of pyrexia (31). An adjuvant control group and animals immunized with HBcAg alone was not included, as it has been previously shown that it does not influence the outcome of challenge (7, 29, 30), although we cannot rule out a minimal adjuvating effect. The experiment was unblinded on day 21 postchallenge. Animals with a Rowland’s ECF index of 6 and above were classified as susceptible to ECF (11, 31). Ten animals, nine in group 7 and one in group 8, were classified as immune to challenge (Fig. 6F, Table VIII). Relative to the control group, which experienced an approximately LD_93_ challenge (the highest used with p67C), the vaccine efficacy in group 7 was calculated to be ∼53%. A robust significant difference was found when comparing the ECF scores from groups 7 and 8 (*p* = 0.004) using linear regression. This effect was unchanged (*p* = 0.003) when including breed and age in the model, as both were not important (*p* = 0.140 and *p* = 0.233, respectively). Moreover, the protection difference between the two groups analyzed by a nonparametric Fisher exact test is also strongly significantly different between the two groups (*p* = 0.005). Parametric logistic regression showed similar significance of difference in immunity (*p* = 0.009) and, again, the breed and age of the animals were of no importance (*p* = 0.994 and *p* = 0.902, respectively) (Table VIII).

**Table VIII.** Summary of protection results and statistical analysis of protection and ECF score differences between groups 7 and 8

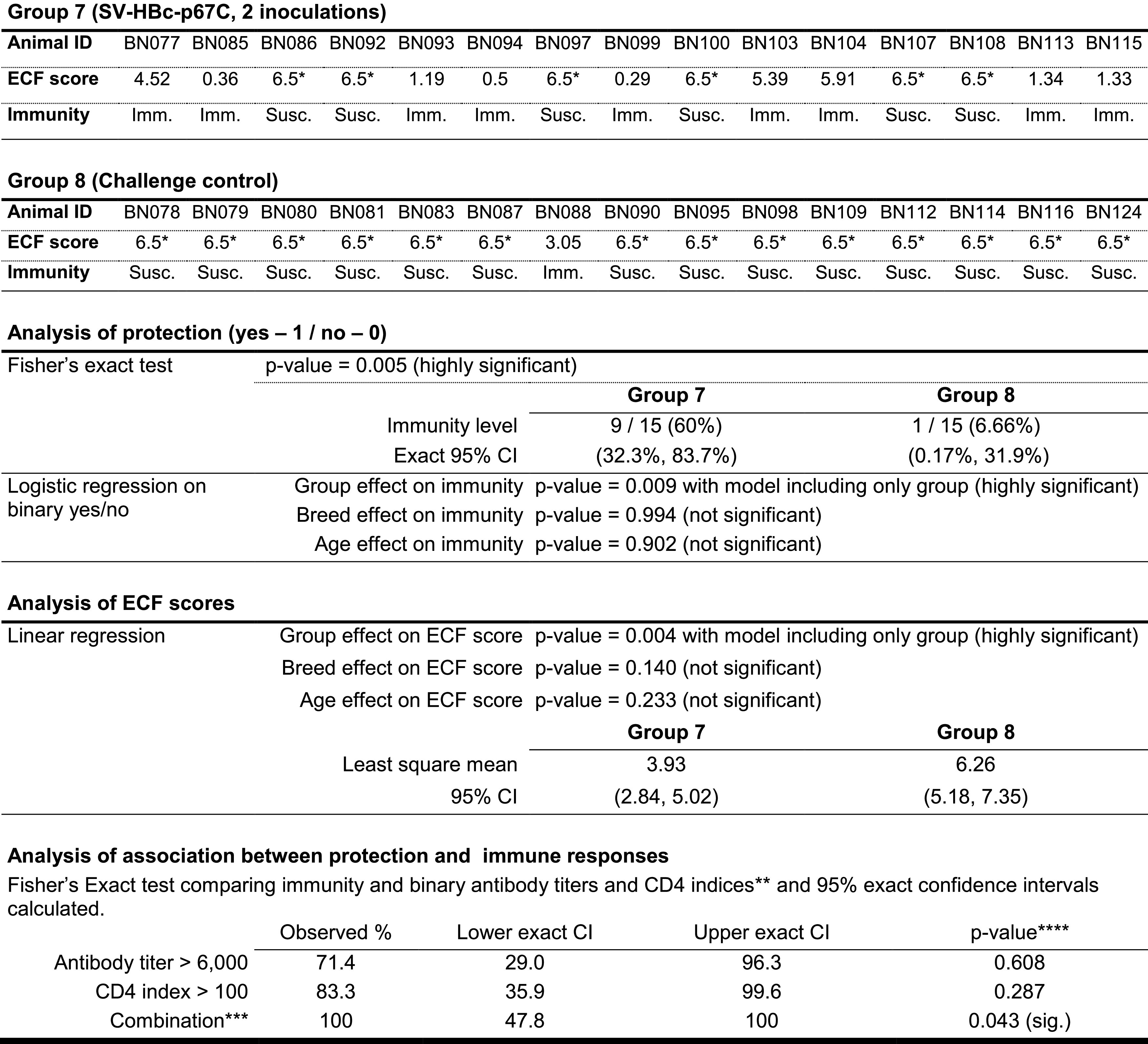

* Final ECF score set to 6.5 (humane end point).

** Half-max Ab titers at day 77 and CD4 proliferation indices at day 77 using 25-mer overlapping peptides as stimuli.

*** Comparison with Ab titer ≤6,000; CD4 index ≤100; and combinaton = low for both or either.

**** Combination of the two parameters in which both thresholds (Ab titer > 6000 and CD4 index > 100) need to be true for protection association.

CI, confidence interval; ID, identifier; Imm., immune; sig., significant; Susc. susceptible.

It is important to highlight one animal in group 8 was classified as immune to ECF because of spontaneous recovery following clinical infection (ECF score of 3.05). This is a well-described phenomenon, as the severity of ECF is sporozoite dose dependent and there is a variation in the threshold of infectivity for individual animals (38, 39). In contrast, six of the ECF immune cattle in group 7 had an ECF score of <1.4 and the other three had a score between 4.5 and 5.99 (Table VIII). Taking into account that ECF is a dose-dependent disease and historically, the maximum challenge dose was an LD_70_ (whereas we are challenging with double the amount of sporozoites to get an LD_93_), and keeping in mind we are using three times less Ag than in previous experiments (7, 10, 11), the combination of SV-HBc-p67C provided an improvement in vaccine efficacy and it is a very promising immunization regimen.

A statistical analysis was performed to assess the association between immunity and Ab titers and CD4 indices individually or in combination. Both parameters individually showed nonsignificant association with immunity, Fisher test *p* = 0.608 for Ab titers and 0.287 for CD4 index. However, in combination, a significant association was observed (*p* = 0.043). Ab titers higher than 6000 indicated a survival rate of 71% (LD_93_ challenge), whereas a CD4 index higher than 100 indicated that 83% of the cattle are immune. When the two previous parameters threshold are true and they are taken in combination (Abs > 6000 and CD4 >100), the level of immunity observed was 100%. Unfortunately, because of the variability in responses among the animals, the confidence intervals for these parameters are large (Table VIII). Although this makes the power of predictability weaker, it indicates the importance of Abs and CD4 responses to protect against ECF. However, further investigations are needed to increase the strength of their association.

## Discussion

The use of nanotechnology in vaccinology has been increasing exponentially in the past decade (40), leading to the birth of “nanovaccinology” (41). Particulate Ags are often more immunogenic than those in a soluble form, and nanotechnology offers an opportunity to design Ag-containing nanoparticles that vary in composition, size, shape, and surface properties to boost the antigenicity of candidate vaccine Ags and modulate immune responses. HBcAg VLPs and 50 nm SV-140–C_18_ SVs are two nanoparticle technologies that are reported to function as superior Ag delivery systems (12, 16). Our objective was to evaluate the capacity of both platforms to improve the immunogenicity and vaccine potential of an 80-aa domain of the p67 Ag from *T. parva* called p67C (8).

In this study, we describe successful generation of HBcAg-p67C VLPs using a glycine-rich linker sequence that flanked p67C cloned into the major immunodominant region of the HBcAg, overcoming the length limitation that the system faced previously with other *T. parva* Ags (22). Conceptually, SV nanoparticles offer a simpler technology as overnight incubation with an Ag results in its adsorption to the SVs and generation of particulate Ag, albeit in a different format to that by VLPs (23, 34). Altering the surface chemistry of SV-140 to create a hydrophobic surface property by adding -C_18_ functional groups to them resulted in optimal adsorption of s-p67C. In contrast, in a study with the E2 Ag of BVDV-1, the nonfunctionalized SVs gave the highest level of Ag adsorption (23), pointing out there is not a gold-standard SV and, therefore, specific Ags must be tested with a variety of SVs to find the optimal nanoparticle.

Assessment of Ag-specific Ab and CD4^+^ T cell responses has been informative on the performance of the individual VLP and SV Ag delivery systems and their intrinsic properties in stimulating different arms of the immune response. HBcAg-p67C VLPs with a *T = 4* symmetry are predicted to contain 120 dimeric subunits and should display 240 copies of p67C (42). It is reasonable to speculate that the display of p67C at high-density and in a regular array on the VLPs results in improved cross-linking of surface Ig and B cell activation (12, 42, 43), resulting in the high p67C Ab response that included a switch to an IgG2 subtype. Unlike in other species, there is no clear evidence for a major difference in the functional role of bovine IgG1 and IgG2. Both subtypes can fix complement, although there are some differences in their opsonizing capacity with superior performance associated with IgG2 (44). Bovine allotypic variants of IgG1, IgG2, and IgG3 have been described (45–48), but reagents to assay them are not available. Thus, little is known about their functional importance. Interestingly enough, the high Ab responses were not in agreement with the CD4^+^ T cell responses in this group of animals, as the cellular responses were directed mainly to the HBcAg polypeptide sequences. Such bias in the cellular immune response to chimeric VLPs has also been described in other studies (14, 49).

The SV-p67C nanoparticle represents an immunologically interesting delivery platform as Ag is adsorbed on the external and internal surfaces of the particle. This interaction is stable and most likely results in creating an Ag depot (50). Priming of an Ab response could occur by direct interaction of B cells with p67C at the surface of the SV or via interaction with soluble Ag that is released from the particles. However, the former, if it occurs, does not promote an Ab switch and the latter may contribute to the delayed peak of the Ab response to the last SV-p67C Ag boost at day 91 instead of day 77, as seen with HBcAg-p67C and s-p67C. Ag bound to the internal surfaces is likely to be protected from degradation and the subsequent endocytosis of the SV-p67C into professional APCs, may facilitate intact p67C entering the MHC class II Ag-processing and presentation pathway via the endosomal system (51), resulting in the strong CD4^+^ T cell response detected in this study. Indeed, using confocal fluorescence microscopy, we demonstrated that the SV-p67C–FITC preparations can be trafficked into an acidic compartment of cells in in vitro experiments. We did not detect CD4^+^ T cell proliferative responses to empty SVs, indicating that the particles do not provide nonspecific stimuli to these cells in in vitro assays. Silica nanoparticles have been shown to activate a proinflammatory cytokine response (52, 53), a pathway also exploited by widely used commercial adjuvants (54, 55). This could contribute in vivo to the enhanced p67C-specific immune responses primed by SV-p67C observed in this study.

The addition of an adjuvant had no effect on the immune response to the E2 Ag of BVDV-1 delivered by SVs to sheep (35) or mice (23) in immunogenicity studies. In these studies, there were no detectable differences in the quantity of the Ab or cellular immune responses primed with or without the use of Quil-A as an adjuvant. In contrast, in the current study, inoculation of cattle with SV-p67C without ISA 206 VG primed very poor Ab and CD4^+^ responses. But the inclusion of the adjuvant ISA 206 VG stimulated a balanced immune response, indicating that the possible enhancing effect of the SVs, is further potentiated by this adjuvant. The capacity of SVs to enhance the immune response may be related to whether the adsorbed Ag is a good or poor immunogen (e.g., the E2 Ag of BVDV-1 and s-p67C polypeptide, respectively). However, it is likely that other factors influenced the outcome. The interaction between the different Ags with the SV nanoparticles may play a critical role, affecting the release properties of the adsorbed Ag from the SV after cellular uptake. Based on this hypothesis, the p67C molecules may be strongly adsorbed to the SV-140–C_18_ particles and the adjuvant ISA 206 VG could act to increase the cell uptake, making both mechanisms indispensable for successfully eliciting an immune response. In contrast, the E2 Ag may be efficiently released from the SVs following cellular uptake and efficiently presented on the cell surface. It may explain why a conventional adjuvant was not required for E2 Ag (35).

To stimulate both high Ab and T cell p67C responses, we inoculated cattle with p67C Ag in the two different nanoparticle technologies of interest to this study. Simultaneous inoculation of HBcAg-p67C and SV-p67C gave rise to balanced immune responses when the immunogens were formulated with ISA 206 VG and injected as two separate inoculations. In contrast, lower immune responses resulted when the two immunogens were formulated as one inoculation. We speculate that destabilization of the VLPs in this formulation leads to loss of p67C immunogenicity. This hypothesis is supported by the differences in the immune parameters we measured in groups 5 and 6 including a higher Ab response to HBcAg in the latter group. However, it remain to be explored the possibility to formulate the two immunogens separately with adjuvant and mix them in one inoculum before injection.

Our observations raised the possibility that coinoculation with that same Ag via two nanoparticle technologies may act synergistically to elicit ideal protective immune responses and provide superior protection to that induced by three inoculations of 450 μg s-p67C with ISA 206 VG, in which ∼50% cattle were immune to an LD_70_ sporozoite challenge (11). Hence, we immunized 15 cattle with three doses of HBcAg-p67C and SV-p67C (equivalent of 140–150 μg of s-p67C per dose) formulated separately. As expected from the immunogenicity studies, the two nanoparticle systems stimulated both p67C-specific Ab and CD4^+^ T cell responses in all 15 cattle, corroborating not only that the two delivery systems did not compete, but they acted synergistically to generate balanced immune responses. This is in line with a previous study in mice, which used Ad5/MERS and boosted with spike protein nanoparticles and they found that balanced Ab and cell-mediated responses were generated (56). Importantly, in the current study, upon challenge, ∼53% of the cattle were immune to an approximately LD_93_ sporozoite challenge, which is the highest challenge dose used to test p67C protective capacity and using more than three times less p67C Ag to date (7, 10, 11). ECF is a dose-dependent disease and the animals in the challenge experiment got double the amount of sporozoites compared with any of the previous published experiments (7, 10, 11), the immune response generated specifically against p67C had to fight a significantly higher parasite load to get 53% of the animals protected. Moreover, the outcome of the challenge experiment allowed an assessment of the association between the Ab titers and the CD4 indices with protection. The association of both parameters with protection individually was NS. However, when the two parameters were taken in combination, the association was significant with an observed level of immunity of 100%, indicating that both immune parameters are associated with protection against ECF. However, further investigations are needed to assess the individual associations of the immune parameters with protection. This is a work in progress and a priority in ECF research.

The findings reported in this study support the combined use of VLPs and SV technology as a novel vaccination strategy to enhance the immunogenicity of otherwise poor Ags in large animal vaccine studies. In the current study, the nanoparticle platforms acted synergistically for enhanced efficacy against a complex parasite like *T. parva*. The simplicity of SVs to adsorb and deliver an Ag without measurable adverse reactions, combined with low manufacturing cost, make them a very attractive platform for Ag delivery. These results also demonstrate the huge potential of the use of VLPs for the future development of improved vaccines against ECF. The immune responses could be further improved to achieve a higher vaccine efficacy with two doses of Ag or the inclusion of other sequences from p67 that contain additional sporozoite-neutralizing sites (9, 10). Moreover, it has been demonstrated that heterologous prime-boost vaccination regimen could enhance the immune response to certain Ags (reviewed in Ref. 57). These are aspects that will be explored in future studies. These nanoparticle technologies have the potential to underpin the development of a more cost-effective and robust vaccine to improve the control of ECF, the most important disease affecting cattle in sub-Saharan Africa.

## Supplementary Material

Data Supplement
