# Supplementary material for: Synergistic Effect of Two Nanotechnologies Enhances the Protective Capacity of the Theileria parva Sporozoite p67C Antigen in Cattle
Source: J Immunol. 2021 Jan 8;206(4):686–99. doi: 10.4049/jimmunol.2000442 (PMC7851744; doi:10.4049/jimmunol.2000442)
Supplement: Data Supplement [file JI_2000442.zip › JI_2000442_Supplemental_Material_1.pdf]

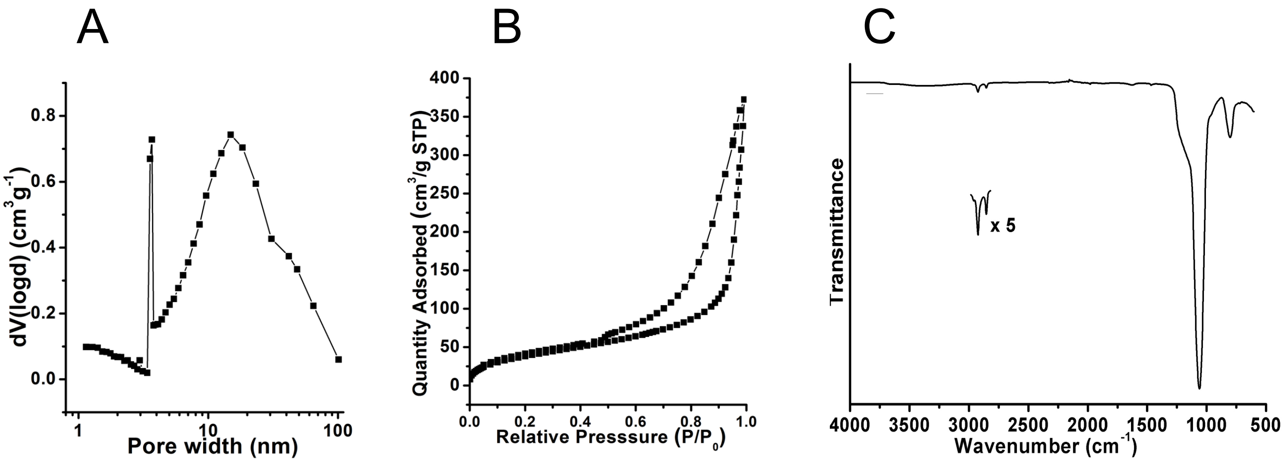

Fig. S1. Characterization of the SV-140-C_{18} nanoparticles. (*A*) BJH pore size distribution curve calculated from the desorption branch of SV-140-C_{18}; (*B*) Total pore volume and Brunauer-Emmett-Teller surface area of SV-140-C_{18} and (*C*) FTIR spectrum of SV-140-C_{18} nanoparticles.

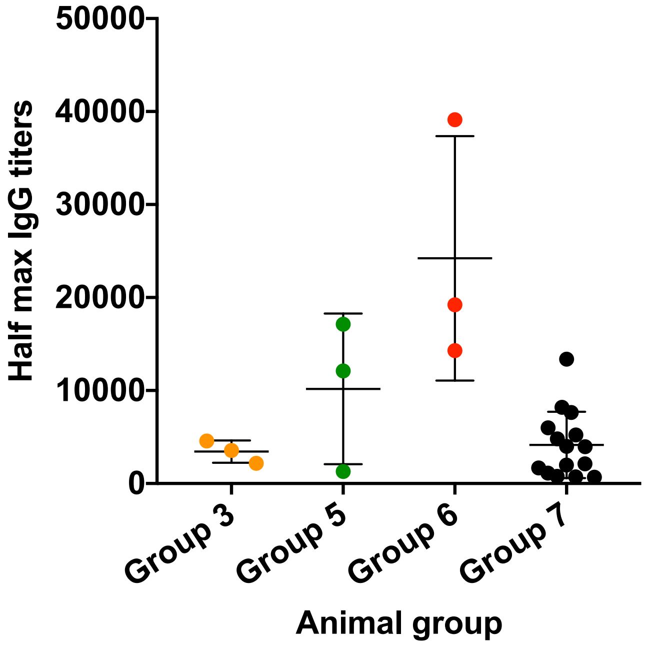

Figure S2. HBcAg specific total IgG antibody levels at day 77 in sera from individual animals inoculated with three doses of: HBcAg-p67C (Group 3, *N*=3), HBcAg-p67C + SV-p67C as two formulations (Group 5, *N*=3), HBcAg-p67C + SV-p67C as one formulation (Group 6, *N*=3) or HBcAg-p67C + SV-p67C as two formulations under the challenge experiment (Group 7, *N*=15). The group average and standard deviation are also shown.

Table SI. 25-mer linear peptides overlapping by 16 amino acids used in the CD4+/CD8+ proliferation assay and ELISpot; and 15-mer linear peptides overlapping in 7 amino acids used in the linear p67C epitope mapping assay.

25-mer linear p67C peptides used for CD4+ IFNγ ELISpot and proliferation assay.

Peptide 1-25	GTGGGSLRGLDLSEEEVKKILDEIV
Peptide 10-34	LDLSEEEVKKILDEIVKDPSDGELG
Peptide 19-43	KILDEIVKDPSDGELGLGDLSDPSG
Peptide 29-53	SDGELGLGDLSDPSGRSSERQPSLG
Peptide 38-62	LSDPSGRSSERQPSLGPSLVITDGQ
Peptide 47-71	ERQPSLGPSLVITDGQAGPTIVSPT
Peptide 56-80	LVITDGQAGPTIVSPTGPTIAAGGE

15-mer linear p67C peptides used for linear p67C epitope mapping.

Peptide 1-15	GTGGGSLRGLDLSEE
Peptide 9-23	GLDLSEEEVKKILDE
Peptide 17-31	VKKILDEIVKDPSDG
Peptide 25-39	VKDPSDGELGLGDLS
Peptide 33-47	LGLGDLSDPSGRSSE
Peptide 41-55	PSGRSSERQPSLGPS
Peptide 49-63	QPSLGPSLVITDGQA
Peptide 57-71	VITDGQAGPTIVSPT
Peptide 65-80	PTIVSPTGPTIAAGG
